# The U-Net Approaches to Evaluation of Dental Bite-Wing Radiographs: An Artificial Intelligence Study

**DOI:** 10.3390/diagnostics13030453

**Published:** 2023-01-26

**Authors:** Oğuzhan Baydar, Ingrid Różyło-Kalinowska, Karolina Futyma-Gąbka, Hande Sağlam

**Affiliations:** 1 Department of Oral and Maxillofacial Radiology, Faculty of Dentistry, Ege University, 35040 İzmir, Turkey; 2 Department of Dental and Maxillofacial Radiodiagnostics, Medical University of Lublin, ul. Doktora Witolda Chodźki 6, 20-093 Lublin, Poland; 3 75. Yıl Oral and Dental Health Center, 06230 Ankara, Turkey

**Keywords:** artificial intelligence, bite-wing radiography, deep learning, segmentation

## Abstract

Bite-wing radiographs are one of the most used intraoral radiography techniques in dentistry. AI is extremely important in terms of more efficient patient care in the field of dentistry. The aim of this study was to perform a diagnostic evaluation on bite-wing radiographs with an AI model based on CNNs. In this study, 500 bite-wing radiographs in the radiography archive of Eskişehir Osmangazi University, Faculty of Dentistry, Department of Oral and Maxillofacial Radiology were used. The CranioCatch labeling program (CranioCatch, Eskisehir, Turkey) with tooth decays, crowns, pulp, restoration material, and root-filling material for five different diagnoses were made by labeling the segmentation technique. The U-Net architecture was used to develop the AI model. F1 score, sensitivity, and precision results of the study, respectively, caries 0.8818–0.8235–0.9491, crown; 0.9629–0.9285–1, pulp; 0.9631–0.9843–0.9429, with restoration material; and 0.9714–0.9622–0.9807 was obtained as 0.9722–0.9459–1 for the root filling material. This study has shown that an AI model can be used to automatically evaluate bite-wing radiographs and the results are promising. Owing to these automatically prepared charts, physicians in a clinical intense tempo will be able to work more efficiently and quickly.

## 1. Introduction

The artificial intelligence (AI) branch, which is called the deep learning method, has been used in the solution of many medical problems in recent years and is a field of study that includes artificial neural networks and similar machine learning algorithms with many hidden layers. The idea that visual diagnosis can be improved using artificial intelligence in radiology to produce lower error rates than the human observer has ushered in an exciting era with clinical and research capabilities [1,2]. The detection and classification of lesions with machine learning, automatic image segmentation, data analysis, the extraction of radiographic features, prioritization of reporting, study triage, and image reconstruction are important technological developments for computer-aided medicine applications. Optimizing AI-related technology, providing reliable diagnostic guidance, assisting radiologists in making clinical diagnosis decisions; reducing the workload of radiologists and saving time for patients and providers; and considering the uneven distribution of medical resources, artificial intelligence-based systems can contribute to reducing barriers, especially in centers without radiologists. Artificial intelligence applications continue to have a huge impact on our quality of life in many sectors [1,2]. AI can produce more sensitive, quick, and reliable results in comparison to a human due to its significant advantages such as mathematical computational power, storage capacity, the ability to perform various tasks, and the ability to be continuously trained in large data sets [2,3]. Artificial neural networks are one of the most crucial building blocks of artificial intelligence, and they express mathematical models that imitate the human brain [4]. These models work by transmitting signals similar to the mechanism of biological neural networks in the human brain [5]. Neural networks have various features that can perform many tasks in the field of medicine and dentistry [6]. For these neural networks to perform more complex and difficult tasks, convolutional neural networks (CNNs) are used. 

CNNs can also be expressed as “deep learning” [7]. Deep convolutional neural networks (DCNN) consist of multi-layer artificial neural networks. As the number of layers increases, the mathematical computation power also increases. Due to these layers, DCNN can process larger data and perform many desired tasks. The success of these networks depends on the quantity and quality of the uploaded training data [7,8,9,10]. 

AI and CNNs have been used in many studies on two-dimensional (2D) and three-dimensional (3D) images [11]. In dental radiology, it has been used in many areas, such as the determination of anatomical landmarks in cephalometric radiographs, tooth identification and numbering, the detection of caries and pathology in the periapical region, the determination of alveolar bone loss in the periodontal region, and the evaluation of the root morphology of teeth [12,13,14,15,16,17]. 

In dentistry, for special diagnoses, apart from panoramic and extraoral radiography techniques, intraoral radiography techniques such as bitewing and periapical radiographs are also preferred frequently. Bite-wing radiography is an intraoral technique that allows for the imaging of the crowns, roots, and a portion of the alveolar bone around the maxillary and mandibular teeth simultaneously. This allows for the viewing and evaluation of more teeth on a single image when compared to periapical radiographs. Bite-wing radiographs help in the detection of interproximal caries, alveolar bone loss, and calculus [18]. In this technique, by giving an 8–10 degrees vertical angle to the beam, the overlapping of the teeth is prevented, and the beam passes through the contact point. Thus, approximal regions are observed more clearly compared to other techniques. Therefore, it is of critical importance in the evaluation of caries in this region [18]. However, the diagnosis of interproximal caries can be difficult despite the use of bite-wing radiographs [19]. Therefore, in recent years, it has been aimed to detect approximal caries with high accuracy, and the demand for computer-aided programs has increased [19,20,21,22].

The aim of the study is to perform a diagnostic evaluation on bite-wing radiographs, to evaluate bitewing images with artificial intelligence applications trained with deep learning methods, and to determine the success and reliability of the developed artificial intelligence.

## 2. Materials and Methods

### 2.1. Patient Selection

Bite-wing radiographs obtained for various reasons, such as approximal caries, the determination of the alveolar bone level, and evaluation of existing restorations, were used in the archive of Eskişehir Osmangazi University, Faculty of Dentistry, Department of Oral and Maxillofacial Radiology. The data set included a total of 500 anonymized bite-wing adult radiographs obtained between November 2018 and January 2020. Only high-quality bite-wing radiographs were included in the study. Radiographs obtained with errors (biting the wing incorrectly, bending of the film, cone-cut, false positioning/angulating, motion artifacts, etc.) were excluded from this study. The study was approved by the Eskisehir Osmangazi University Faculty of Medicine Clinical Research Ethics Committee (approval number: 33, 15 June 2021), and the principles of the Declaration of Helsinki were followed.

### 2.2. Radiographic Dataset

For imaging, ProX periapical X-ray unit (Planmeca, Helsinki, Finland) was operated at 220–240-V, 60 kVp, 2 mA, and 0.05 s scan time, and a focus receptor distance was 20 cm. All digital bite-wing radiograph images were acquired with storage phosphor plates (SPPs) of the ProScanner Phosphor Plates and Scanning System (Planmeca, Helsinki, Finland) with imaging dimensions of 44.1 × 30.4 mm, pixel size of 30 µm, and resolution of 12 lp/mm. The project was created by saving the radiographs in jpeg format and uploading them to the CranioCatch (Eskisehir, Turkey) labeling software.

### 2.3. Image Evaluation

**Figure 1:**
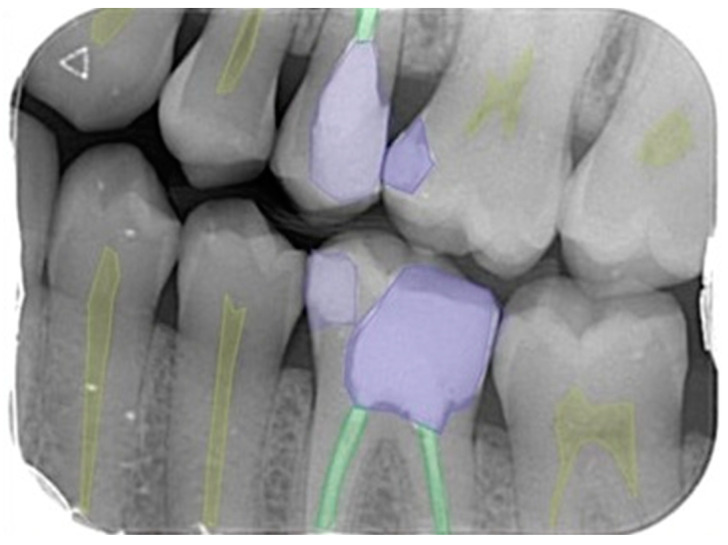
Polygonal style on bite-wing radiography image; labeling for 5 different dental diagnoses as dental pulp, dental caries, dental restorative filling material, dental crown, and dental root canal filling material.

Labeling is the process of identifying areas in an image and determining which region the object belongs to. The labeling of 5 different dental diagnoses (dental caries, dental crown, dental pulp, dental restorative filling material, and dental root canal filling material) was performed by a 3-year experienced research assistant and an 11-year-experienced oral and maxillofacial radiologist using the segmentation technique via the CranioCatch (CranioCatch Eskisehir, Turkey) software for all images (Figure 1). After determining the outer borders of the dental restorations and pathologies evaluated by the segmentation method, the images were stored in the JSON format on the software’s cloud-based server.

### 2.4. Deep Convolutional Neural Network 

**Figure 2:**
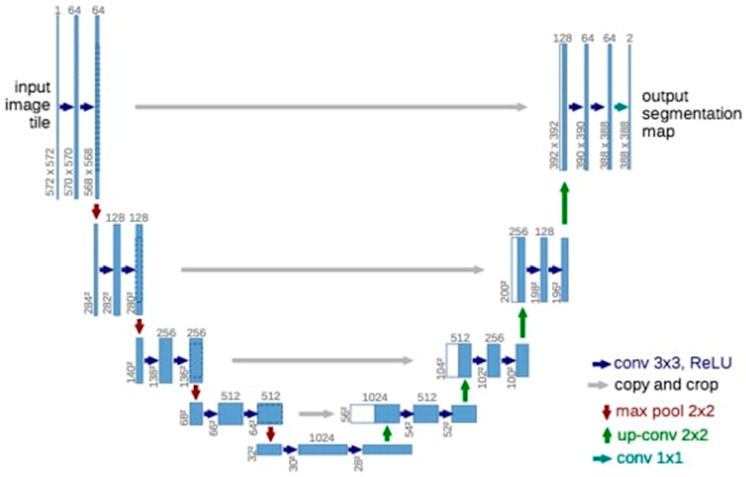
The 3D U-Net architecture. Blue boxes represent feature maps. The number of channels is denoted above each feature map.

Deep learning was performed using the U-Net model implemented with the PyTorch library (version 1.4.0). The U-Net architecture was used for semantic partitioning tasks (Figure 2) [23]. Our encoder-decoder type consists of four block levels, including two convolutional layers. There is a max pool layer in the encoding part and up-convolutional layers in the decoding part. Each block has 32, 64, 128, or 256 convolutional filters. The layer consists of 512 pleated filters. Jump links from the encoding layers to the corresponding layers in the decoding part are used.

### 2.5. Model Pipeline

The PyTorch library was used in this study. The PyTorch library is an open-source library that aims to remove this barrier for both researchers and practitioners. The Python open-source programming language (v.3.6.1; Python Software Foundation, Wilmington, DE, USA) and PyTorch library were used for model development. In our study, model training was performed on a computer equipped with 16 GB RAM and NVIDIA GeForce GTX 1660 TI graphics card.

### 2.6. Training Phase

Approximately 80% of the data set we have is divided into three parts, including training, 10% testing, and 10% validation (Table 1). The training of the AI model was performed using 200 epochs. The learning rate of the model was determined as 0.0001. The data from the testing group were not reused. 

### 2.7. Statistical Analysis

The confusion matrix was used to evaluate model performance. This matrix is a meaningful table that summarizes the predicted situation with the actual situation.

### 2.8. Metrics Calculation Procedure

The metrics used to evaluate the success of the model were as follows:

- True positive (TP): dental diagnoses correctly detected and segmented.
- False positive (FP): dental diagnoses detected but incorrectly segmented.
- False negative (FN): dental diagnoses incorrectly detected and segmented.

The performance metrics of the created models were calculated using TP, TP, and FN values as follows:

- Sensitivity (Recall): $\frac{\mathrm{TP}\;}{\mathrm{TP}\;+\;\mathrm{FN}}$
- Precision, positive predictive value (PPV): $\frac{\;\mathrm{TP}\;}{\mathrm{TP}\;+\;\mathrm{FP}}$
- F1 score: $\frac{2\;\mathrm{TP}\;}{2\mathrm{TP}\;+\;\mathrm{FP}\;+\;\mathrm{FN}}$

Intersection over Union (IoU): Intersection over union (IoU) is a standard evaluation method implemented in Pascal VOC 2012 using true positives, false positives, and false negatives. The IoU metric shows the overlapping region between the result of the proposed method and the exact reference space (ground truth) [24]. In this study, this area was accepted as TP (true positive) if it was greater than 50% and FP (false positive) if it was smaller.

## 3. Results

**Figure 3:**
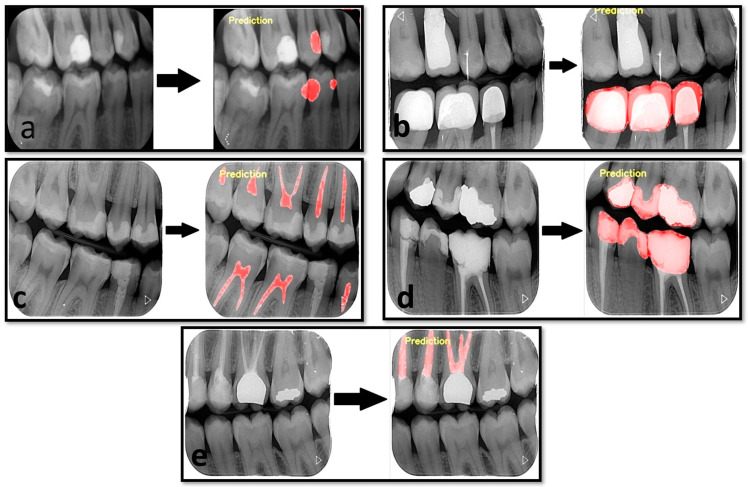
Estimated segmentation images created by AI model. (**a**). Estimated image for dental caries. (**b**). Estimated image for the dental crown. (**c**). Estimated image for a dental pulp. (**d**). Dental restoration filling material. (**e**). Dental root-canal filling material.

The results of our study showed that the developed artificial intelligence model was successful in its predictions (Figure 3). Although the least success value was in the diagnosis of caries, a high success rate of over 95% was obtained for other dental diagnoses. Sensitivity values were between 0.8235–0.9843, while precision values were between 0.9429–1 for the five determined dental labels. The F1 score ranged from 0.8818 to 0.9722 (Table 2). 

The F1 score, sensitivity, and precision results of the study were, respectively, dental caries; 0.8818–0.8235–0.9491, dental crown; 0.9629–0.9285–1, dental pulp; 0.9631–0.9843–0.9429, with dental restoration filling material; and 0.9714–0.9622–0.9807 was obtained as 0.9722–0.9459–1 for the dental root-canal filling material (Table 2).

## 4. Discussion

Dental radiographs enable dentists to identify conditions that are impossible to identify and detect in the clinic. Panoramic, cephalometric, hand-wrist, periapical, occlusal, and bite-wing radiographs are routinely used for diagnostic purposes in the dentistry clinic. Panoramic radiography offers an overview of the teeth and jaws, tooth deficiencies, dental caries, periodontal problems, impacted teeth, etc. It is an extraoral imaging method that provides the information necessary for the diagnosis of many problems. Periapical, occlusal, and bite-wing radiographs are intraoral imaging methods that can obtain higher-resolution images. With these radiographs, the teeth and surrounding tissues can be easily evaluated. 

The bite-wing radiography technique, also known as the interproximal technique, is a technique in which portions of teeth in the maxilla and mandible can be obtained on a single film. The tooth root and apex are not seen in bite-wing radiographs. With this method, the diagnosis of interproximal caries that are difficult to detect and secondary caries that occur under restorations are diagnosed in extra-oral radiography images [25]. AI has been used in the evaluation of dental radiographs in a limited number of studies and generally on two-dimensional radiographs. Using this method, many dental problems, such as missing teeth, dental crowns, dental caries, periodontal problems, and periapical pathologies, have begun to be detected with artificial intelligence applications trained with deep learning algorithms. There are various AI studies that have used a bite-wing radiography technique for the dental diagnosis process [18,26,27]. 

Bayrakdar et al. aimed to evaluate the effectiveness of CNNs in the diagnosis of approximal carious lesions, and 1000 bitewing radiography images were used. In the model trained with the YOLO (you only look once) model, the positive predictive value in the test group was found to be lower (86.56%) compared to our study, despite the higher amount of bite-wing radiography images used [28]. García-Cañas et al. evaluated the performance of CNNs across four different models using three hundred bite-wing radiography images. According to the results of their studies, it was seen that the sensitivity was between 41.6 and 87%, and the positive predictive value was between 42 and 88.6% [29]. On the other hand, Devlin et al., on the other hand, evaluated their ability to detect 65 caries lesions previously diagnosed on 24 bite-wing radiographs among two groups of dentists with and without artificial intelligence support. They reported that the use of artificial intelligence increased the ability to detect enamel-level approximal caries by 71% [30]. Devito et al. evaluated the effect of an artificial intelligence model application on the radiographic diagnostic capacity of approximal caries, using bite-wing radiographs of ex vivo teeth to confirm the clinical and histopathological diagnosis of caries. The results of their study showed that when the diagnostic performance of clinicians and the neural network model was examined by receiver operating characteristic (ROC) analysis, it significantly increased the diagnostic capacity of approximal caries [31].

Cantu et al. [32] also developed a model based on the deep learning method in bite-wing radiographs and performed the detection of dental caries lesions. In their studies, 3686 bite-wing radiographs were evaluated by four experienced dentists, and dental caries lesions were marked as pixels. They performed deep learning by applying a U-Net model. As a result, the artificial intelligence model they developed showed higher success with 80% accuracy compared to the dentists, who made 71% correct predictions in the study. Sensitivity was higher in neural networks compared to dentists (0.75 vs. 0.36). In addition, while the sensitivity values were significantly different between the two groups, the difference between the positive predictive values of the groups (0.70 vs. 0.75) was not significant. The F1 score, on the other hand, was almost twice that of the dentists in neural networks and differed significantly [32]. 

Bayrakdar et al. [33] dental caries detection and segmentation were performed using VGG-16 and U-Net architectures on bite-wing radiographs. They compared the performance of physicians with five different experiences with the AI model. They reported that the AI model outperformed the research assistants. They stated that the F1 score of the AI model was 0.78 in dental caries detection and 0.81 in dental caries segmentation [33]. 

Srivastava et al. [34] worked on an automatic system for the detection of dental caries. They used over three thousand bite-wing radiographs in their studies and developed a system for the automatic detection of dental caries. The AI system they developed consisted of a deep, fully convolutional neural network (FCNN). They reported that this automatic system outperformed dentists in terms of sensitivity (80.5 vs. ~41.7) and F1 score (70 vs. ~53.3) [34]. In these studies, the fact that artificial intelligence models found higher accuracy than dentists actually had supportive results for the views that the models could minimize human-induced errors.

U-Net architecture, which is a CNN method, is one of the most successful methods in image segmentation on medical images. The U-Net architecture consists of encoding and decoding parts, the encoding process consists of a VGG-style CNN model, and the decoding occurs by the iterative application of up-convolution to the feature channel [35,36]. Conversely, the U-Net architecture segmentation can be successfully performed on images using a limited amount of training data. Therefore, in our study, we used U-Net architecture, which is frequently used in image segmentation in the medical field [37].

Lee et al. [27] detected dental caries on bite-wing radiographs with a CNN model they developed with U-Net architecture. A total of 304 bite-wing radiographs were used. The researchers reported that the F1 score for the diagnostic performance of the CNN model was 64.14%, the precision was 63.29%, and the recall was 65.02%. They stated that this deep learning model could guide the physician in the diagnosis process [27]. In this study, U-Net architecture was used as a deep learning architecture. The F1 score, sensitivity, and precision values of the AI model in dental caries detection and segmentation in our study were 0.8818–0.8235–0.9491, respectively [38]. In our study, we think that reasons such as the increased number of data, the use of data replication parameters, and segmentation differences may have increased the success of the model.

Moran et al. [38], on the other hand, used Inception and ResNet architectures, which have different architectural structures than U-Net architectures in the detection of dental caries on bite-wing radiographs. They reported that they obtained the best accuracy rate of 73.3% with the Inception model in their study, in which they used 112 bite-wing radiographs. The most important feature that distinguishes the Inception architectures developed by the GoogleNet team from other architectures is that they use filters that can perform the same task at a smaller size instead of making large convolutions. Thus, the number of transactions made is reduced. The most important feature of ResNet is the jumping process between layers. This process is called ‘ResBlock’. Thus, even if nothing is learned in the previous layer, the information in the old layer is applied to the new layer, making the model a stronger architecture [39]. The difference in the Inception v2 model, which is another version of GoogleNet Inception architectures, from other Inception architectures is the size and number of convolution filters used. Thus, the computational load of the architecture is reduced [40]. 

Yasa et al. [26] worked on a model based on a deep learning method. In the study, tooth detection and numbering were performed on bite-wing radiographs. They included 1125 bite-wing radiographs in their study. They used a pre-trained GoogleNet Inception v2 faster R-CNN network for preprocessing the training data sets by transfer learning, and Tensorflow was used for model development. This developed automatic system correctly identified and numbered 697 out of 715 teeth in the study [26]. When the existing studies in the literature were evaluated, there was one study in which the detection and segmentation of dental diagnoses were made with a deep learning approach using intraoral radiography images. According to Khan et al. [41], in their study, they compared the diagnostic performance of four different deep learning architectures for the diagnosis of dental caries, alveolar bone recession, and inter radicular radiolucencies. As a result, they determined that the U-Net architecture and another deep learning system derived from this architecture gave the most successful results [37]. In this study, we used the U-Net architecture to develop our artificial intelligence model. Our study is the first to perform detection and segmentation using CNNs for different dental diagnoses on bite-wing radiography images.

The success of a developed artificial intelligence model is determined by the formulation of true positive/negative and false positive/negative values. The F1 score obtained by considering all of these values gives information about the success of the model. When we evaluated the F1 score of the models in this study, we obtained values of 0.88 for caries and above 0.95 for other models. Bayrakdar et al. [33], when the F1 scores for the artificial intelligence models developed in that study with five different levels of physicians were evaluated, found that the artificial intelligence model had a better score than the assistant physicians. In that study, the F1 score of the model was evaluated with both object detection and segmentation, and the segmentation method that we used in our study was more successful. In that study, the F1 score of the model was 0.81, while that of the assistant physicians was 0.62 and 0.74 [33]. In this study, the F1 score for the caries detection of the model was found to be higher compared to the literature. However, models should be developed by trying larger data sets, different CNN architectures, and methods in order to increase the F1 score above 0.95 for caries detection in further studies.

## 5. Conclusions

Radiographs, which are routinely used in dental practice, are difficult to interpret and require clinical experience. However, even experienced dentists may miss various problems in radiological examination. The artificial intelligence branch, which is called the deep learning method, has been used in the solution of many medical problems in recent years and is a field of study that includes artificial neural networks and similar machine learning algorithms with many hidden layers. The results of our study show that the success of artificial intelligence algorithms in bite-wing radiography images is promising. The success rate is predicted by future studies using similar larger data sets, and it is thought that these automatic systems used for the diagnosis will save time and enable dentists and specialists to work more efficiently in the clinic.

## Figures and Tables

**Table 1:** Training, testing, and validation groups on bite-wing images for five different dental diagnoses.

Bite-Wing Images			
Diagnoses	Training Group	Testing Group	Validation Group
Dental Caries	1052	33	132
Dental Crown	264	9	36
Dental pulp	1560	50	200
Dental restoration filling material	1308	41	164
Dental root-canal filling material	812	25	100

**Table 2:** The value of AI model estimation performance measurement using the confusion matrix.

Measurement Value			
	Sensitivity (Recall)	Precision	F1 Score
Dental caries	0.8235	0.9491	0.8818
Dental crown	0.9285	1	0.9629
Dental pulp	0.9843	0.9429	0.9631
Dental restoration filling material	0.9622	0.9807	0.9714
Dental root-canal filling material	0.9459	1	0.9722

## Data Availability

Not applicable.
